# Activation of MAPK and FoxO by Manganese (Mn) in Rat Neonatal Primary Astrocyte Cultures

**DOI:** 10.1371/journal.pone.0094753

**Published:** 2014-05-02

**Authors:** Vernat Exil, Li Ping, Yingchun Yu, Sudipta Chakraborty, Samuel W. Caito, K. Sam Wells, Pratap Karki, Eunsook Lee, Michael Aschner

**Affiliations:** 1 Department of Pediatrics, Thomas P. Graham Division of Pediatric Cardiology, Monroe Carrell Jr. Children's Hospital, Vanderbilt University School of Medicine, Nashville, Tennessee, United States of America; 2 Department of Pediatrics, Division of Pediatric Toxicology, Monroe Carrell Jr. Children's Hospital, Vanderbilt University School of Medicine, Nashville, Tennessee, United States of America; 3 Molecular Physiology and Biophysics, Vanderbilt University School of Medicine, Nashville, Tennessee, United States of America; 4 Department of Physiology, Meharry Medical College, Nashville, Tennessee, United States of America; University of Louisville, United States of America

## Abstract

Environmental exposure to manganese (Mn) leads to a neurodegenerative disease that has shared clinical characteristics with Parkinson's disease (PD). Mn-induced neurotoxicity is time- and dose-dependent, due in part to oxidative stress. We ascertained the molecular targets involved in Mn-induced neurodegeneration using astrocyte culture as: (1) Astrocytes are vital for information processing within the brain, (2) their redox potential is essential in mitigating reactive oxygen species (ROS) levels, and (3) they are targeted early in the course of Mn toxicity. We first tested protein levels of Mn superoxide dismutase -2 (SOD-2) and glutathione peroxidase (GPx-1) as surrogates of astrocytic oxidative stress response. We assessed levels of the forkhead winged-helix transcription factor O (FoxO) in response to Mn exposure. FoxO is highly regulated by the insulin-signaling pathway. FoxO mediates cellular responses to toxic stress and modulates adaptive responses. We hypothesized that FoxO is fundamental in mediating oxidative stress response upon Mn treatment, and may be a biomarker of Mn-induced neurodegeneration. Our results indicate that 100 or 500 µM of MnCl_2_ led to increased levels of FoxO (dephosphorylated and phosphorylated) compared with control cells (P<0.01). p-FoxO disappeared from the cytosol upon Mn exposure. Pre-treatment of cultured cells with (*R*)-(−)-2-oxothiazolidine-4-carboxylic acid (OTC), a cysteine analog rescued the cytosolic FoxO. At these concentrations, MAPK phosphorylation, in particular p38 and ERK, and PPAR gamma coactivator-1 (PGC-1) levels were increased, while AKT phosphorylation remained unchanged. FoxO phosphorylation level was markedly reduced with the use of SB203580 (a p38 MAPK inhibitor) and PD98059 (an ERK inhibitor). We conclude that FoxO phosphorylation after Mn exposure occurs in parallel with, and independent of the insulin-signaling pathway. FoxO levels and its translocation into the nucleus are part of early events compensating for Mn-induced neurotoxicity and may serve as valuable targets for neuroprotection in the setting of Mn-induced neurodegeneration.

## Introduction

Environmental exposure to manganese (Mn) leads to manganism, characterized by dopaminergic (DAergic) neurodegeneration, with clinical findings in humans that are reminiscent of idiopathic Parkinson's disease (PD) [1]. Mn exposure is often occupationally related (i.e. in welders and miners) [2]. Although a small amount of Mn is essential for a variety of normal cellular processes [3], toxic levels of Mn in humans may lead to major health concerns.

The present study is related to the possible implications of Mn effects in brain cells, as Mn-neurotoxicity may have a role in neurodegenerative processes and disorders of the basal ganglia. The prevalence of PD and other neurodegenerative diseases are on the rise, and prevalence and incidence of PD in the general population is perhaps well underestimated [4], [5], [6], [7]. An etiologic role for Mn and other toxicants in the sporadic form of PD has been often evoked, as only 10% of PD cases can be ascribed to genetic factors [5], [8]. Scientific studies addressing a possible role for Mn in PD are timely, as interaction between genetics and environmental factors may lead to PD [7], [9]. The discovery that intravenous drugs contaminated with 1-methyl-4-phenyl-1,2,3,6-tetrahydro-pyridine (MPTP) led to clinical signs of PD in humans is only recent [10], [11], [12]. It is now evident that other toxicants are involved in PD-like cases. Prolonged occupational exposure to Mn is linked to an increased risk for PD [2], [13], [14]. Patients with Mn-induced signs of PD show symptoms at an earlier age [2]. The clinical presentation includes rigidity, tremor, dystonia and bradykinesia [1], [2]. Regions of the brain that are affected by Mn include the nigrostriatal pathway, predominantly the globus pallidus, causing nigrostriatal dysfunction [15], [16]. Dysfunction of the mitochondria is a shared hallmark of both PD and Mn exposure [5], [8], [17], [18], leading some to hypothesize that PD and manganism should be considered as disorders of mitochondrial energy deficiency. At the molecular level, Mn leads to increased levels of reactive oxygen species (ROS) triggering neuronal cell death [19], [20], [21], [22], [23], [24]. Mn also leads to alteration in energy and glucose metabolism with ATP depletion and cell death [25], [26], [27], [28], [29], concomitant with mitochondrial dysfunction.

Most cells, including astrocytes, have protective mechanisms against ROS [30]. Molecules that prevent excessive ROS generation or protect against prooxidants are emerging as research targets for aging, cardiovascular illnesses, cancer, obesity, diabetes and other chronic illnesses [31]. Antioxidant genes are highly regulated in time and space. FoxO, a forkhead box transcription factor class O, is critical for cell survival as it is involved in transcriptional regulation of antioxidant genes [32]. Putative elements of this transcriptional regulatory complex include PPAR gamma coactivator-1 (PGC-1) [33]. Studies on the role of FoxO and PGC-1 in Mn-induced neurotoxicity have yet to be reported.

Herein, we set out to determine FoxO-related biological targets associated with Mn exposure in cultured neonatal astrocytes. We show that Mn treatment (both at 6 and at 24 hrs) led to induction of mitochondrial superoxide dismutase 2 (SOD2), but no significant changes in glutathione peroxidase (GSH peroxidase, GPx-1). We also show that F2-isoprostane (F2-IsoP) levels, biomarkers of ROS generation and lipid peroxidation, were elevated in response to Mn exposure. Both FoxO 3a (phosphorylated and total) and PGC-1 protein levels were coordinately elevated at 6 and at 24 hrs after Mn exposure. In this paper, we explore a Mn-induced phosphorylation mechanism of FoxO in astrocytes. We tested whether it occurs through the insulin-signaling pathway and AKT or MAPK activation. Our data lead us to propose a protective and compensatory role for FoxO as a biomarker of neurodegeneration. We also propose a role for MAPK activation in FoxO signaling as a contributing effector in Mn-induced neurodegeneration. Pre-treatment of cultured cells with (*R*)-(−)-2-oxothiazolidine-4-carboxylic acid (OTC), a cysteine analog and a precursor of GSH, the major intracellular antioxidant, attenuated the levels of Mn induced F2-IsoP, and further increased total FoxO protein expression. OTC also led to changes in the subcellular localization of the FoxO proteins, reversing the subcellular changes induced by Mn exposure. These data suggest that regulation of FoxO may serve as potential therapeutic targets for Mn-induced neurodegenerative processes.

## Methods

### Isolation and Culture of the Neonatal Astrocytes

We used primary neonatal astrocyte cultures from Sprague-Dawley rats. Astrocytes were isolated based on the method initially described by Barger and Basile, with minor modifications [34]. One-day-old newborn pups were used. After initial plating, the cell medium was changed twice per week. Cells consistently reached confluence in 14 days. The purity of the cultures (assessed by the expression of glial fibrillary acidic protein; GFAP) was greater than 95%. Protocols for these studies were reviewed and approved by the Vanderbilt University Institutional Animal Care and Use Committee (IACUC).

### Mn treatment of Cultured Astrocytes

Cultured astrocytes were exposed to Mn^2+^ (as MnCl_2_). MnCl_2_ was obtained from Sigma (St. Louis, MO) and cells were exposed for 6 or 24 hrs. The following two Mn concentrations were used, 100 and 500 µM (as MnCl_2_), in all the experiments. In other sets of experiments, cells were pre-treated with (*R*)-(−)-2-oxothiazolidine-4-carboxylic acid (OTC) for 24 hrs at a concentration of 1 mM, (referred to as OTC pretreated cells). OTC was obtained from Sigma (St. Louis, MO, Cat. #, 06254). Upon completion of the timed Mn exposures, the cells were rinsed with PBS and harvested for protein isolation and purification.

### Western Blot Analysis

Whole cell lysates were prepared from astrocyte cultures after Mn treatment with RIPA lysis buffer, as previously described [35], [36]. Twenty-five µg of total protein per lane was run on 10% SDS PAGE gels and transferred to nitrocellulose membrane. For western blot analysis, the following primary antibodies were obtained from Santa Cruz Biotechnology: SOD-2 (SOD-2, SC-18508), glutathione peroxidase (GPx-1, SC-22146), and phospho-FoxO 3a (p-FKHRL1, SC-101683,). We obtained total FoxO 3a (FoxO 3a) from Cell Signaling (75D8). The PGC-1α antibody was custom made and previously published by our group [37]. AKT was obtained from cell signaling (Cat# 4685) and P-MAPK/P-ERK from Promega (Cat # V803A). ERK1/2 pAb, pan antibody that recognizes both phosphorylated and non-phosphorylated ERK1/2 was also from Promega (Cat # V1141). Both phospho-p38 (Cat #4215) and total p38 (Cat #9212) antibodies were from cell signaling. LY 294002 (Cat# L-7962), an inhibitor of phosphatidylinositol 3-kinase (PI3K), was obtained from Calbiochem. LY 294002 leads to inactivation for Akt/PKB. SB 203580 (Cat# S-3400), a selective inhibitor of p38 MAPK, was obtained from LC Laboratories (Woburn, MA). PD98059, an ERK inhibitor, was obtained from Selleck Chemicals (Cat #S1177).

### Measurement of Isoprostane Species (F2- IsoPs)

After Mn exposure, astrocytes were harvested and placed at −80°C until use. F_2_-IsoPs levels were measured with an isotope dilution method and detected with gas chromatography/mass spectrometry (GC/MS), following standard protocols for purification and analysis, as previously described [38].

### Immunocytochemistry Imaging

For the immunocytochemistry staining, we assessed expression of pFoxO and total FoxO in four different experimental groups of cultured astrocytes: (1) untreated (control cells), (2) MnCl_2_ treatment alone for 6 hrs, (3) OTC pretreatment for 24 hrs prior to exposure to MnCl_2_ for 6 hrs, and (4) OTC alone. Astrocytes were fixed in cold methanol and incubated on ice for 5 minutes. They were then placed at room temperature. Blocking was performed for 2 hrs at room temperature, using Power Block Universal Blocking Reagent (from BioGenex, Freemont, CA, Cat #HK085). The primary antibodies, FoxO (from Cell Signaling, see above) and p-FoxO (from Santa Cruz Biotechnology, Inc., see above), were used at a dilution of 1∶50. All primary antibody dilutions were prepared in a diluent from BioGenex (from BioGenex, Freemont, CA, cat HK156-5k). Incubation of all primary antibodies was carried out for 2 hrs at room temperature. Secondary antibodies (Alexa Fluor 568 goat anti-rabbit IgG_H+L_) were prepared in the above-mentioned BioGenex diluent at a concentration of 2 µg/µl. Incubation of all secondary antibodies was also carried out at room temperature for 2 hrs. Finally nuclear staining was performed at room temperature for 20 minutes, using 1 µM of DAPI (Life Technologies, D3571). After washing in DDH_2_O, coverslips were mounted onto glass-slides with mounting medium (Aqua-Poly/Mount, Polysciences, Inc). High-resolution imaging was performed in the Cell Imaging Shared Resource Center at Vanderbilt University Medical Center.

### Statistical Analysis

Data presented in this paper are reported as Mean ± S.D. of the mean. One-way analysis of variance (ANOVA) followed by Bonferroni's *post hoc* test was performed for analyzing differences between treatment groups. Statistical significance was considered for P values <0.05.

## Results

### *Evidence of ROS accumulation and a role for* (*R*)-(−)-2-oxothiazolidine-4-carboxylic acid (OTC) in early and late Mn exposure

**Figure 1:**
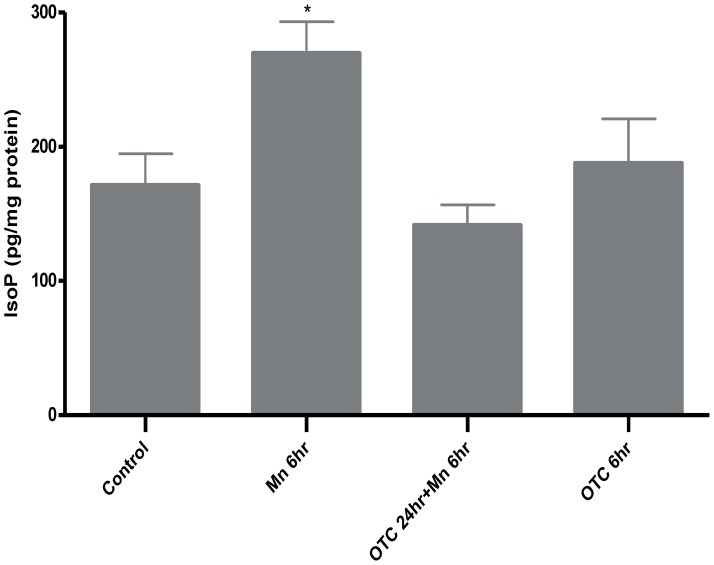
OTC attenuates Mn-induced increased F2-Isoprostane levels in astrocytes. The histogram represents changes in values for Isoprostane levels in pg/mg of protein with Mn treatment in the presence or absence of OTC. F2-IsoP levels were significantly elevated after 6 hrs of 500 µM of Mn exposure alone. Pre-treatment with 1 mM of OTC for 24 hrs markedly reduced the effect of high doses of Mn. OTC alone did not lead to significant changes in F2-IsoP levels in astrocytes. Data was collected from N = 4 replicates. Isoprostane levels are expressed as Mean ± SD, * p<0.05.

First, we assessed the stress signals underlying Mn exposure and oxidative stress response by measuring astrocytic F_2_-IsoP levels. We found that F_2_-IsoP levels increased in response to Mn exposure. IsoP levels were 171.5±23.19 pg/mg protein for control cells, compared with 269.9±23.19 pg/mg protein after 6 hrs in Mn exposed astrocytes (Fig.1). F2-IsoP levels in astrocytes pre-treated with OTC followed by Mn exposure for 6 hrs were statistically indistinguishable from controls. OTC treatment alone did not cause changes in F2-IsoP levels. F2-IsoP levels in OTC pre-treated astrocytes for 24 hr followed by 6 hours of MnCl_2_ exposure were 141.7±15.02 pg/mg protein. In astrocytes treated with OTC alone F2IsoP levels were 187.9±32.93 pg/mg protein (Fig. 1).

**Figure 2:**
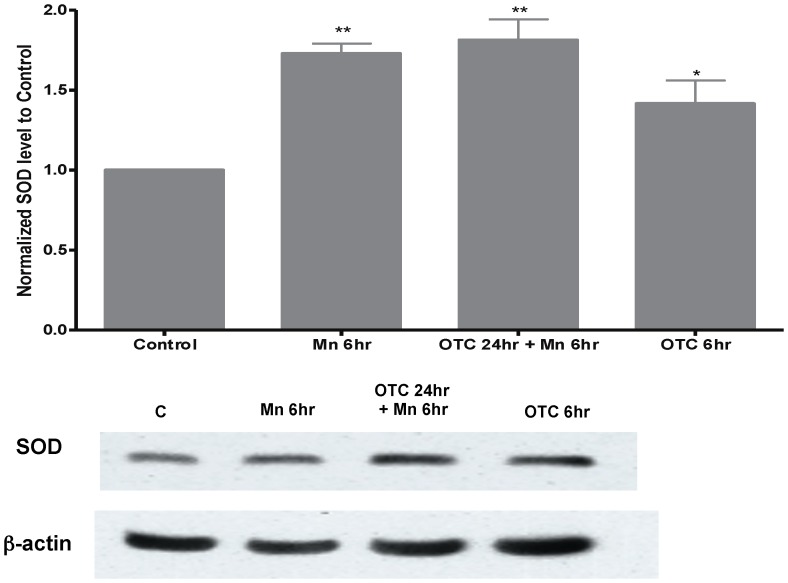
SOD-2 levels in Mn-treated neonatal astrocytes. This histogram is representative of fold changes in densitometry for SOD-2 protein levels using Western blot analysis. Each column represents a normalized ratio (fold-changes) to β-actin and to control. SOD-2 levels increased significantly after 6 hrs of 500 µM Mn treatment. OTC pre-treatment in the presence of Mn led to further elevation of SOD-2 levels, in a statistically significant manner. OTC alone also led to changes in SOD-2 levels, but the difference or fold-change was not significant from SOD-2 levels from control cells. Data was collected from N = 5 replicates (*p<0.05, **p<0.01).

**Figure 3:**
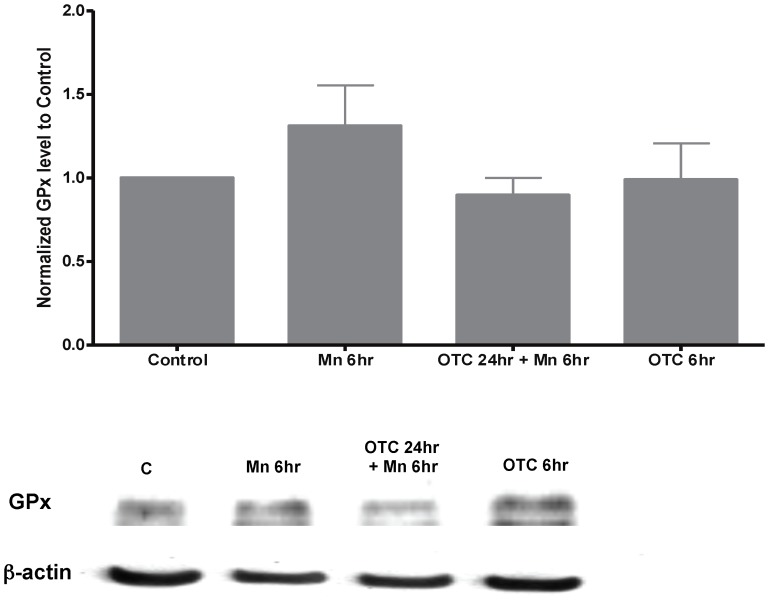
Glutathione peroxidase (GPx) levels in Mn-treated astrocytes. This is a representative histogram of fold-changes in GPxlevels by densitometry, using Western blot analysis. There was a trend for elevated levels of GPx levels after 6 hrs of 500 µM of Mn exposure. The fold-changes observed for GPx-1 were not significant. Similarly, OTC alone or OTC pretreatment followed by Mn exposure did not lead to any changes in GPx-1 protein levels. These data were collected from N = 5 replicates similar to the biological samples used to measure SOD-2 fold changes. Results were independently normalized to β-actin and to control prior to statistical analysis.

We subsequently tested whether the changes in F2-IsoP levels in Mn exposed astrocytes correlated with oxidative stress response. We measured protein levels of SOD-2 and GPx-1. We found that levels of SOD-2 were elevated after 6 hrs of Mn exposure (Fig. 2). SOD-2 protein levels increased 1.73±0.06 fold after Mn exposure (Fig. 2). Pre-treatment of these cells with OTC (for 24 hrs) prior to Mn exposure (for 6 hrs) led to further elevation of SOD-2 (1.8±0.13). Interestingly, OTC alone also increased SOD-2 levels by 1.4±0.15 fold (Fig. 2). On the other hand, Mn exposure increased GPx-1 levels by 1.3±0.2 fold, compared to the controls with no statistical significance (Fig. 3). OTC pre-treatment of these astrocytes in the presence or absence of Mn did not lead to changes in GPx1 levels (Fig 3).

### Phosphorylated FoxO3a is significantly induced by Mn exposure

**Figure 4:**
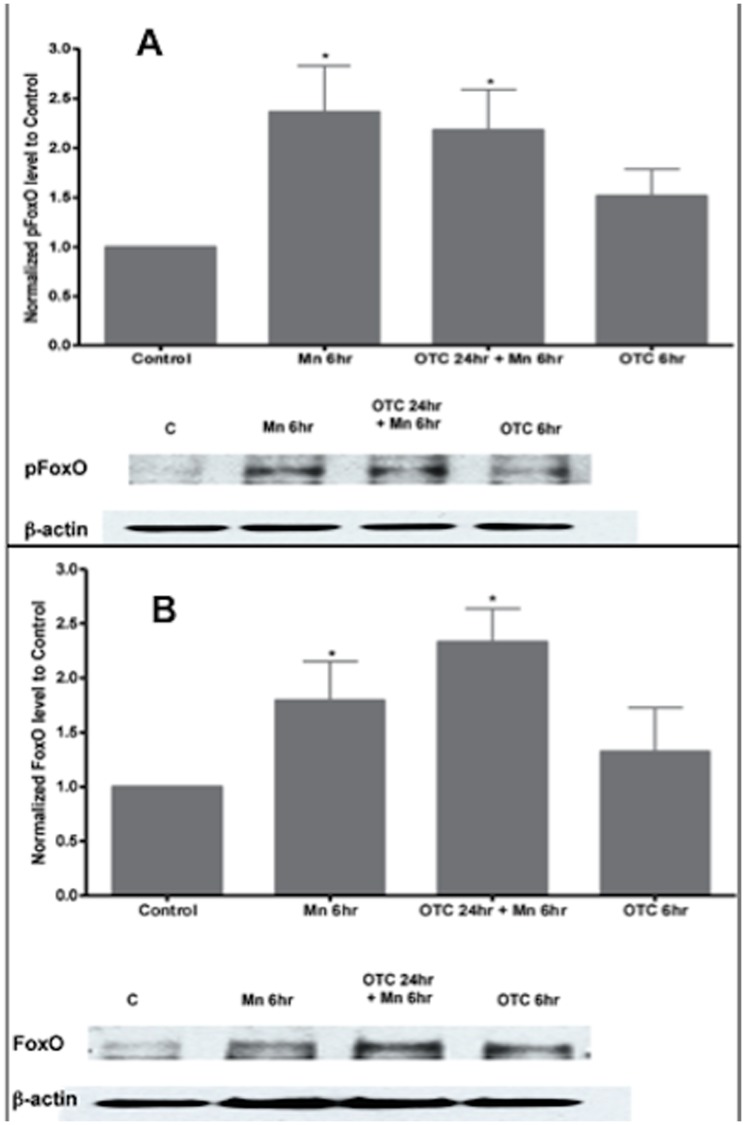
Mn treatment leads to elevated levels of FoxO in cultured astrocytes. (A) Western blot analysis of phosphorylated FoxO (pFoxO) in Mn-treated astrocytes. These histograms are representative of fold-changes measured by densitometry for phosphorylated and total FoxO protein levels, using Western blot analysis. (A) Levels of phosphorylated FoxO were significantly increased with 500 µM of Mn treatment (p<0.05). However, OTC led to only slight reduction in pFoxO in Mn-treated astrocytes. There were no changes in FoxO levels with OTC alone. (B). Western blot analysis of total FoxO (FoxO) protein levels in Mn-treated astrocytes. The histogram in Figure B is representative of fold-changes in total FoxO levels. Note that total FoxO also increased in response to Mn treatment. Data collected for these experiments is from N = 5 replicates. There was a significant increase when the cells were treated with OTC prior to Mn treatment, *p<0.05.

Mn exposure led to a concentration- and time-dependent increase in the levels of both phosphorylated and total FoxO (Fig. 4A and 4B). After 6 hrs of Mn exposure, there was a 2.4±0.5 fold increase in phosphorylated FoxO (pFKHRL1). Pre-treatment with OTC (for 24 hrs) prior to Mn exposure also led to a 2.2±0.4 fold increase in phosphorylated FoxO levels. OTC alone also increased phosphorylated FoxO by a 1.5±0.3 fold (Fig 4 A). There was a similar increase in total FoxO in these cells, with a 1.8±0.4 fold increase in total FoxO (FoxO) with Mn treatment (Fig 4B, bar-graph 3). There was a further 2.4±0.3 fold increase in total FoxO levels in the presence of OTC and Mn. OTC alone caused a 1.3±0.4 fold change in total FoxO levels (Fig 4B).

### MAPK activation and AKT levels with Fox0 phosphorylation and Mn exposure

**Figure 5:**
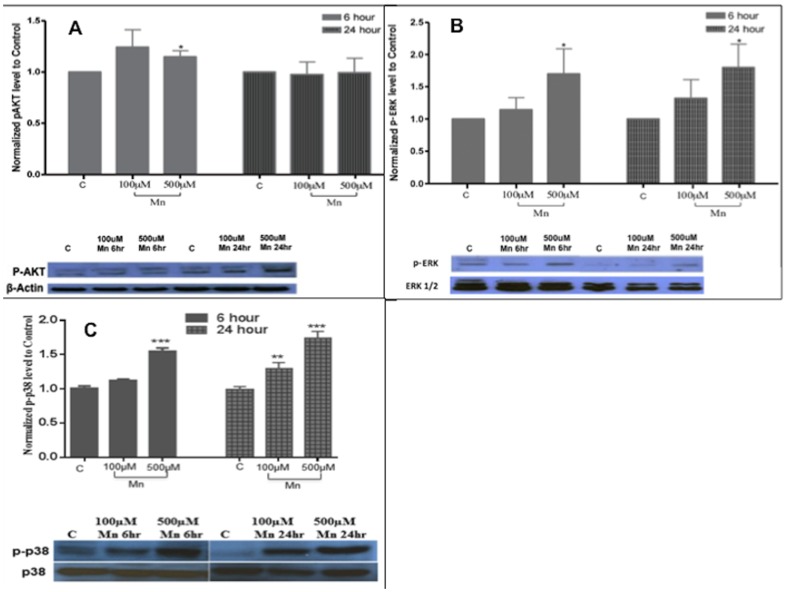
MAPK but not AKT are activated in Mn treated astrocytes. Figures A,B and C are representative histograms reporting changes in phospho AKT(p-AKT),phospho ERK (p-ERK) and phosphor p38 (p-p38) levels. There was only minimal increase in p-AKT levels after Mn treatment. After 6 hrs of Mn exposure AKT levels were 1.3±0.22 with 100 µM and 1.19±0.06 with 500 µM. Similarly levels of AKT essentially unchanged after 24 hrs of Mn treatment, as they were 0.97±0.12 with 100 µM and 0.99±0.14 with 500 µM. Next, we assessed phosphorylated ERK1/2 levels (Fig 5A). At 6 hrs after Mn treatment, the fold-changes in pERK1/2 levels were 1.14±0.19 and 2.2±0.4 for 100 and 500 µM Mn, respectively. After 24 hrs of Mn treatment, the fold-changes in pMAPK levels were 1.32±0.3 and 1.8±0.35 for 100 and 500 µM Mn, respectively. This was significant with p<0.01 Fig.5B). At 6 hrs of Mn (500 µM) exposure, phospho p38 levels were increased to 1.55±0.14 fold and at 24 hrs the fold increase were1.31±0.16 and 1.74±0.14 for 100 and 500 µM of Mn, respectively (Fig.5C). Data analysis for all phosphorylation measurements are from Western blot analysis of N = 5 replicates. These fold-changes are being reported as normalized ratios to both corresponding total protein and to untreated controls, *p<0.05, ***p<0.001. Although notable changes were observed in pERK levels with 100 µM of MnCl_2_, these changes were not statistically significant.

Given that phosphorylated FoxO protein levels were markedly increased with Mn exposure, next we assessed both AKT and MAPK levels, as putative molecular signals upstream of FoxO phosphorylation. Phosphorylated AKT levels were not significantly changed with Mn exposure (Fig. 5A). After 6 hrs of Mn exposure, AKT levels were 1.3±0.22 with 100 µM of Mn and 1.19±0.06 with 500 µM of Mn. Similarly levels of AKT essentially did not change after 24 hrs of Mn treatment, as they were 0.97±0.12 with 100 µM and 0.99±0.14 with 500 µM. Next, we assessed phosphorylated MAPK (in particular, ERK and p38) levels. Mn exposure led to a time- and concentration–dependent increase in MAPK/ERK phosphorylation. After 6 hrs of Mn exposure, the fold-changes in phospho-MAPK/ERK levels were 1.14±0.19 and 2.2±0.4 for 100 and 500 µM Mn, respectively. After 24 hrs of Mn treatment, the fold-changes in phospho-MAPK/ERK levels were 1.32±0.3 and 1.8±0.35 for 100 and 500 µM Mn, respectively (p<0.01, for 500 µM of Mn) (Fig.5B). Likewise, Mn also increased the phosphorylation of MAPK/p38, another MAPK, in a time and concentration dependent manner (Fig. 5C). After 6 hrs of Mn exposure, only 500 µM of Mn increased phospho-p38 levels significantly (1.55±0.14 fold increase), but after 24 hrs of Mn exposure, both 100 and 500 µM of Mn increased the phosphorylation of p38 significantly (Fig. 5C). The increment in phospho-p38 levels after 24 hrs of Mn exposure was 1.31±0.16 and 1.74±0.14 folds for 100 and 500 µM of Mn, respectively. These fold-changes in phospho-MAPKs at 6 and 24 hrs are normalized to both corresponding total MAPK and controls (i.e. untreated cells).

**Figure 6:**
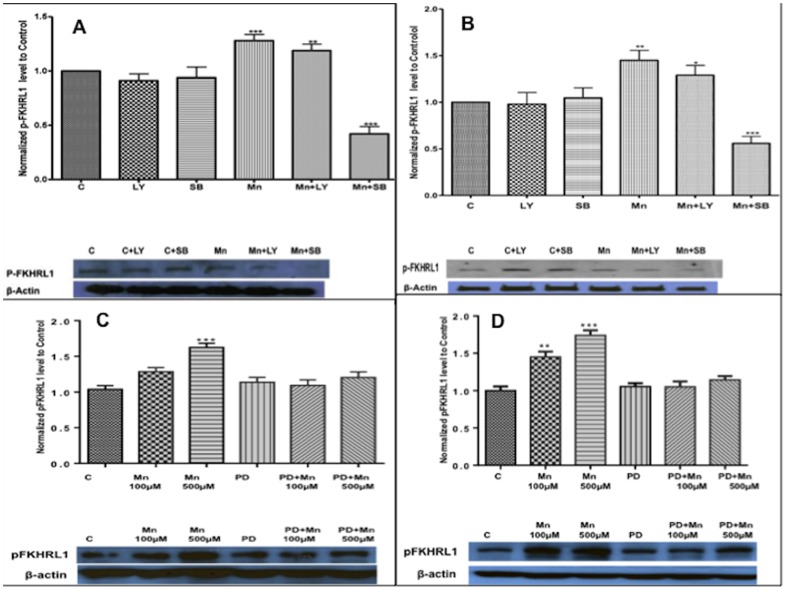
Changes in levels of phosphorylated FoxO (pFoxO) in the presence or of AKT, p38 and ERK inhibitors, LY,SB and PD, respectively. The histograms depict fold-changes is pFoxO levels 6 and 24 hrs after Mn treatment (A and B, C and D respectively). There was increased levels of pFoxO protein levels with Mn treatment. Astrocytes were pre-treated with inhibitors, SB (10 µM), PD (50 µM) or LY (10 µM), for 30 min prior to adding Mn. Mn treatment was done in a time and dose-dependent manner. Note there are 6 hrs of Mn treatment in figure 6A and 6C and 24 hrs of Mn treatment in Figure 6B and 6D. Both in the 6 hrs set and in the 24 hrs set, SB, PD and LY alone did not lead to any changes in pFoxO levels. Data collection is from N = 4 replicates. After Mn treatment, only SB or PD and not LY led to significant changes or reduction in pFoxO levels, *p<0.05, **p<0.01,***p<0.001.

Next, we measured both phosphorylated levels in the presence and absence of their specific inhibitors. LY 294002 was used as an inhibitor of the PKB/AKT insulin-signaling pathway. Given that we observed the increased phosphorylation of both MAPK/ERK and MAPK/p38 by Mn exposure, we used PD98059 (PD) and SB 203580 (SB) to inhibit MAPK/ERK and MAPK/p38 pathways, respectively. Astrocytes were pre-treated with each inhibitor for 30 min prior to Mn exposure. Pre-treated astrocytes with LY, SB or PD alone did not lead to changes in pFoxO levels (Fig 6A–D). Upon Mn exposure, LY pre-treated cells did not alter the levels of phosphorylated FoxO compared to the control cells (Fig 6A and 6B, comparing bar-graphs 4 and 5 of each Figure). However, astrocytes pretreated with SB reduced levels of phosphorylated FoxO after 6 or 24 hrs of Mn exposure (Fig. 6A and 6B, comparing bar-graphs 4 and 6 of each figure). Similarly, pretreatment of astrocytes with PD also attenuated Mn (100, 500 µM)-induced phosphorylated FoxO levels (Fig. 6C and 6D).

### Induction of PPAR gamma coactivator-1 (PGC-1) with manganese exposure

**Figure 7:**
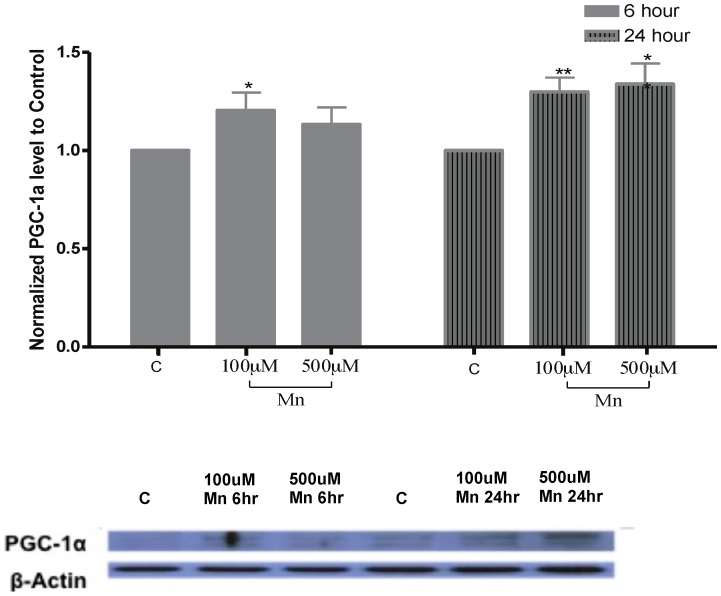
Mn induces PGC-1 levels in astrocytes. This histogram is representative of changes in astrocytic levels of PGC-1 upon Mn exposure for 6 and 24 hrs. The most significant changes were noted 24 hours after Mn treatment. PGC-1 protein levels after 6 hrs of Mn treatment were 1.2±0.09 with 100 µM and 1.13±0.09 with 500 µM of Mn. After 24 hrs of Mn treatment PGC-1 levels were 1.30+/−0.07 with 100 µM of Mn and 1.34+/−0.11 with 500 µM of Mn. Data collection is from N = 4 replicates, *p<0.05, **p<0.01.

Given recent reports that cooperation between FoxO and PGC-1 are required for regulation of oxidative protective genes [39], we tested whether levels of PGC-1were also induced by Mn exposure. Western blot analysis showed that PGC-1 protein expression levels after 6 hrs of Mn exposure were 1.21±0.09 with 100 µM and 1.13±0.09 with 500 µM, compared to the normalized control value as 1. Similarly, after 24 hrs to Mn exposure, PGC-1 levels were 1.30±0.07 with 100 µM and 1.34±0.11 with 500 µM (Fig. 7). Changes in PGC-1 levels were more pronounced after 24 hrs of Mn exposure. These changes were also statistically significant at low Mn concentrations after 6 hrs (100 µM), whereas Mn exposure for 24 hrs significantly increased PGC-1 levels at both 100 and 500 µM (P<0.01 and P<0.05, respectively). Of note, this increased levels of PGC-1 protein expression occurred simultaneously with changes in FoxO levels. These data suggest that both FoxO and PGC-1 might act in concert to regulate protective mechanisms for cell survival in Mn treated astrocytes.

### Mn leads to nuclear translocation of FoxO in astrocytes

**Figure 8:**
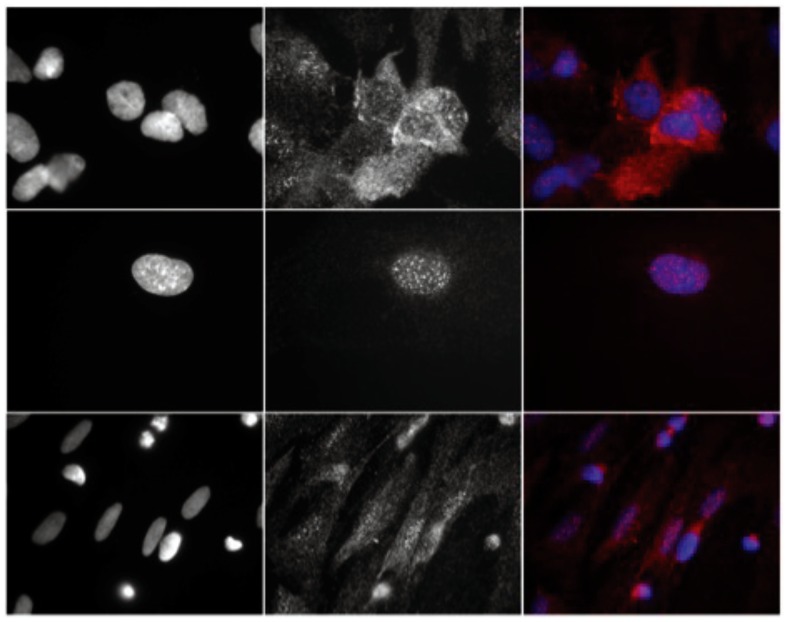
Confocal microscopy images of pFoxO staining in Mn-treated astrocytes in the absence or presence of OTC. Top Row shows control cells stained for nuclei (left column) and pFoxO (middle column), and merged (right column, blue nuclei and red pFoxO). Middle row is cells treated with Mn alone. Note that the cytosolic signals are markedly reduced indicating reduction in the level of the pFoxO epitope in the cytosol. This is in sharp contrast with data suggesting that pFoxO levels are elevated at this time (Figs 4A, 6A, and 6B). This may indicate translocation pFoxO into the nucleus for ROS protecting gene regulation. The bottom row is representative of cells treated with Mn after pretreating with OTC. These cells depict rescue of pFoxO cytosolic levels or return of pFoxO signals to the cytosolic portion of the cell. N = 3 biological replicates in these experiments.

Using immunocytochemistry, we subsequently assessed the subcellular localization of FoxO in Mn-treated and non-treated astrocytes at 6 and 24 hrs, using 100 and 500 µM of MnCl_2_ (as described above). The histological findings were identical for all treated arms of the study. Cell imaging showed that phosphorylated FoxO levels in the cytosol were significantly reduced upon Mn exposure (Fig 8, Middle Row). OTC pre-treatment of astrocytes led to partial recovery of cytosolic pFoxO (Fig 8, Bottom Row). Given that protein level of both phosphorylated FoxO and total FoxO were elevated in the same cell population (Fig 4A, B), these data suggest that manganese toxicity leads to translocation of FoxO species into the nucleus. This translocation is presumed for the purpose of downstream activation of protective genes.

## Discussion

### Manganese exposure and oxidative stress injury in cells

Environmental exposure to Mn is a public health concern, as excessive levels of Mn may lead to encephalopathy and reduced levels of dopamine in the brain [40]. Clinically, Mn toxicity presents extrapyramidal symptoms that resemble PD [41]. Mn accumulates in several brain structures, including the substantia nigra, but mainly in the globus pallidus and other basal ganglia structures. Mn toxicity leads to neurodegeneration, but the mechanism is complex and remains poorly understood [42]. Just as in PD, the prevalent hypotheses of Mn-induced neurodegeneration include mitochondrial dysfunction and oxidative damage [28], [43]. In this study, we explore a role for FoxO as a protective marker and a role for MAPK activation as a contributing effector in Mn-induced neurodegeneration.

Our findings reveal that Mn treated astrocytes display an early and prolonged elevation in oxidative stress markers, characterized by elevated levels of isoprostane (F2-IsoPs) and elevated levels of the antioxidant enzyme SOD-2. These results are in agreement with data previously published by our laboratory [38]. These results also emphasize, as previously published by others, the critical role of astrocytes in glutathione (GSH) metabolism and oxidative stress responses [44]. One rationale for using astrocytes in these experiments is the putative interdependence between astrocytes and neurons in neurodegenerative diseases [45]. There is a putative intermediary role for astrocytes in neurotoxicity caused by various metals [46]. Possibly through paracrine mechanisms, astrocytes can be either protective or detrimental to neighboring cells (neurons), as they are also targets of toxicants that may ultimately lead to neuronal cell death. Given that neuronal response to oxidative stress injury is often damaging and irreversible, efforts for pharmaceutical targets to prevent astrocyte toxicity may yield profitable results. Healthy astrocytes may have a beneficial role in a variety of other neurological injuries [19], [44], [45], [47], [48].

### FoxO induction is critical in early Mn exposure

Mn toxicity leads to elevated levels of FoxO (i.e. both total and phosphorylated FoxO). Downstream of the insulin signaling pathway lays a group of Forkhead subfamily of transcription factors, referred to as the FoxO group. Under conditions of low (basal) protein kinase B (PKB) activity, FoxO protein is predominantly nuclear, where it interacts with essential cofactors for transcriptional activation of genes with context-dependent effects on cellular stress [32], [49], [50], [51]. Upon phosphorylation, PKB translocates to the nucleus and phosphorylates FoxO, resulting in its release from the DNA [52]. This results in a equilibrium shift of FoxO towards the cytosol and repression of the transcription of genes associated with protection against oxidative stress, such as MnSOD-2, GSTs, catalase [32], [53], genes involved in DNA repair (*Gadd45*), cell-cycle arrest (*p27kip1*), and anti-apoptotic effects (*Bim* and *Fas* ligand) [54], [55], [56].

In our studies, both total levels and phosphorylated FoxO levels were elevated after exposure to Mn (Fig 4A, 4B, 6A, and 6B). Dephosphorylated FoxO species would be found mostly in the nucleus (post nuclear translocation), thus serving as one of the determinants for transcription regulation of the antioxidant/protective enzyme genes after exposure to Mn. Similarly, phosphorylated FoxO would be mostly found in the cytosol. Herein, data from cell imaging showed that phosphorylated FoxO levels in the cytosol were significantly reduced with Mn treatment, but cytosolic FoxO can be rescued with OTC (Fig 8). In the setting of elevated levels of both phosphorylated FoxO and total FoxO with Mn treatment (Fig 4A, B), translocation into the nucleus for further downstream reactions is presumed. Our results are in agreement with previously published data suggesting that FoxO regulates mammalian genes in response to oxidative stress [57], [58], [59]. Although activities of the FoxO proteins are also regulated by oxidative stress [58], we conclude from these data that Mn leads to activation or de-phosphorylation of FoxO, causing FoxO to translocate to the nucleus regulating protective antioxidant genes.

### Induction of MAPK and not AKT is involved in early Mn exposure

Our data suggest that this level of Mn-induced FoxO phosphorylation is accomplished in parallel with the insulin-signaling pathway. We found that Mn led to marked increase in MAPK/ERK phosphorylation levels, while the levels of phosphorylated AKT remained relatively unchanged (Fig 5A and 5B). Taking advantage of several individual kinase inhibitors, we were able to further assess the specific involvements of the AKT and MAPK related pathways. We were able to show that inhibitors of MAPKs (MAPK/ERK and MAPK/p38) phosphorylation, but not AKT inhibitor, had a greater role in FoxO phosphorylation levels. From these data, it is possible that Mn exposure can also suppress FoxO activation (over time or at higher doses). There is an elevated level of p-FoxO and compensatory translocation of pFoxO into the nucleus (Fig. 8), but parallel mechanisms for FoxO phosphorylation are triggered through MAPK phosphorylation, in a regulatory feedback, independent of the insulin-signaling pathway. In an unbalanced state where MAPK activation would be prevalent and persistent, FoxO in a predominantly phosphorylated state would not be good for the cell. Therefore, our results suggest that MAPK activation in early Mn exposure is one of the contributing elements in the chain of events that lead to cell death.

Our data suggests that Mn exposure leads to elevated levels of phosphorylated MAPK/ERK and MAPK/p38 (Fig 5B and 5C), which is an activated form of the molecules. Remarkably, OTC led to further elevation of FoxO levels, Inhibition of MAPK phosphorylation led to marked reduction on p-FoxO levels (Fig 6A and 6B), further supporting a key role for MAPK activation in Mn –induced neuronal cell injury. MAP kinases are serine/threonine kinases that are rapidly activated in response to growth factor stimulation. Using astrocyte culture, work previously done in our laboratory showed that Mn leads to MAPK/ERK activation and collapse of the mitochondrial action potential [17]. This MAPK/ERK family includes ERK1 and ERK2. The activated MAPK can translocate to the nucleus to regulate transcription factors. In our model, MAPK activation and the use of antioxidants have opposite roles after Mn exposure. This finding, although new in relation to Mn-induced neurodegeneration, has been reported in other diseases [60], [61], [62]. For example, the MAPK pathway has been shown activated in Alzheimer's disease (AD) patients. Biochemical findings in AD include evidence of increased neuron inflammation, tau hyperphosphorylation, and abnormalities in Aβ trafficking [60]. Inhibition of the MAPK pathway has been proposed to be a target for therapy in insulin resistance and AD [60], [61]. In our model, given that Mn activated both MAPK/ERK and MAPK/p38, we inferred that the changes of FoxO phosphorylation were possible due to activation of these two MAPKs. MAPK/JNK, is another MAPK that was not tested in the present study. However, we cannot exclude its involvement in Mn-induced phosphorylation of FoxO, as others have previously reported a possible MAPK/JNK signaling in FoxO metabolism [63], [64]. Our results demonstrated that inhibitions of MAPK/p38 as well as MAPK/ERK led to marked reduction in pFoxO levels (Fig. 6). These results are in agreement with recent work by Taylor et al suggesting that MAPK/p38 might be contributing to the pathogenesis in Huntington's disease [62]. Taylor and his co-workers proposed the use of MAPK inhibitors to improve Huntington's disease outcome [62]. Our results also suggest that inhibition of the MAPK signaling may be beneficial in the setting of Mn-induced neurodegeneration.

### Coordinated elevation of PGC-1 and FoxO levels may have biological significance with Mn exposure

Our data also suggest that Mn increases the levels of PGC-1α in astrocytes. PGC-1 protein levels were increased parallel with elevated levels of FoxO (Fig. 6A, 6B, and 7). Data showing the protective role for PGC-1α in oxidative stress and neurodegeneration has been previously reported, as PGC-1 deleted animals were shown more sensitive to neurodegenerative effects of 1-Methyl-4-phenyl-1,2,3,6-tetrahydropyridine (MPTP) [33]. The role for cooperation between those two molecules has been recently hypothesized. They act as critical co-regulators of oxidative stress genes [65]. Our data show that both molecules are induced concurrently with Mn exposure. However, it is unknown whether FoxO and PGC-1 act as co-regulators of transcriptions in these settings. Our findings, however, are supportive of previously published data, suggesting that FoxO has a selective role in regulation of protective genes and regulation of cellular mechanisms for cell health and longevity in other organisms [66].

## Conclusion

Environmental exposure to Mn leads to a neurodegenerative illness that has shared pathophysiology and clinical findings with PD. It is hypothesized that increased oxidative stress and elevated ROS levels are at the basis of Mn-induced neuronal toxicity. The molecular intermediates leading to neuronal cell death remain unknown. Given their critical role in maintaining brain homeostasis, using astrocytes for the experiments is relevant to study FoxO and FoxO targets in Mn-induced neurotoxicity. We found that FoxO, MAPK, and PGC-1 were key intermediary molecules in Mn-induced oxidative stress response. Mn treatment induced elevated levels of total FoxO, with reduction of cytosolic pFoxO levels as seen by immunocytochemistry. Mn exposure also triggered MAPK/ERK and MAPK/p38 activations, leading us to believe that the observed phosphorylation or de-activation of FoxO occurred through MAPK, in parallel with the insulin-signaling pathway. Interestingly, phosphorylated MAPK induction was readily corrected with the use of antioxidants, showing simultaneous reduction in levels of phosphorylated FoxO but marked increase in total FoxO (probably mostly dephosphorylated and nuclear in localization). Mn also led to elevated levels of PGC-1, a molecule hypothesized to co-regulate oxidant protective genes with FoxO [39]. We conclude that these key intermediary molecules are part of the biological early responses associated with putative protective mechanism upon Mn-induced neurotoxicity. These changes may have implications for both the Mn-neurotoxicity field and for disorders of the basal ganglia. They may represent valuable targets for protection from Mn-induced neurodegeneration, as mechanisms that lead to reduced levels of MAPK activation (MAPK inhibitors), elevated levels of dephosphorylated FoxO, and elevated levels of PGC-1 should facilitate antioxidant responses.
